# Once-Weekly Mosnodenvir as Dengue Prophylaxis in a Human Infection Model

**DOI:** 10.1056/NEJMc2516395

**Published:** 2026-10-15

**Authors:** Liesbeth Van Wesenbeeck, Freya Rasschaert, Kristen K. Pierce, Guillermo Herrera-Taracena, Beulah P. Sabundayo, Annemie Buelens, Patricia Lutton, Veerle Van Eygen, Megan McKnight, Kim De Clerck, Isabel Fetter, Xi Fang, Nele Goeyvaerts, Kathleen Callewaert, Sarah Sampson, Jeffrey Mayfield, Oliver Lenz, Sandra De Meyer, Thomas N Kakuda, Helen He, Emérito Amaro-Carambot, Ruxandra Draghia-Akli, Marya Carmolli, Marnix Van Loock, Stephen S. Whitehead, Anna P. Durbin

**Affiliations:** 1 Johnson & Johnson, Beerse, Belgium; 2 University of Vermont College of Medicine, Burlington, VT; 3 Johnson & Johnson, Horsham, PA; 4 Johns Hopkins Bloomberg School of Public Health, Baltimore; 5 Johnson & Johnson, San Diego, CA; 6 National Institute of Allergy and Infectious Diseases, Bethesda; 7 Johnson & Johnson, Spring House, PA

## To the Editor:

Dengue, a significant global health threat, currently lacks effective antiviral treatments.^1^ Mosnodenvir is an antiviral against dengue virus (DENV) infection which has reached phase 2 clinical development in a prophylactic setting.^2^ Initial results from this randomized, double-blind, placebo-controlled Phase 2a trial (Cohort 1[C1]) have been published^1^ and were expanded with additional daily (QD) and weekly (Q7D) dose regimens in Cohort 2 (C2). Loading doses (LD) started on Day (D) −2 twice daily (BID) for 2 days, followed by maintenance doses (MD) as QD or Q7D for 21 days (QD, 800 mg/250 mg, Q7D high-dose 450 mg/1200 mg and low-dose 250 mg/500 mg). The under-attenuated virus rDEN3Δ30 was subcutaneously injected on D1 with safety, pharmacokinetics, virology and serology parameters evaluated up to D120 (Figure 1A). The protocol and Supplementary Appendix are available with the full text of this letter at NEJM.org.

The findings in C1 QD high-dose regimen (60% [6/10] without signs of DENV-3 infection [undetectable DENV-3 RNA; no anti-DENV IgM/IgG/nAbs])^2^ were also seen in C2 QD (67% [4/6] without signs of DENV-3 infection) (Figure 1B). These results extended to C2 Q7D regimens with 83% (5/6) without signs of DENV-3 infection in each arm versus 0% (0/6) in the placebo arm. Among participants with available NS4B sequencing data, emerging amino acid variations in NS4B were detected in 4/4 mosnodenvir recipients (A19L [N=1], V91G [N=2], L94F [N=1], Y99H [N=1]) and 2/6 placebo recipients (S60I [N=1] and I201V [N=1]). Mosnodenvir plasma concentrations rapidly increased with the LD and were maintained over the MD phase (Figure 1C and D). No significant safety concerns were identified (Table S1).

These data improve our understanding of mosnodenvir’s dose-dependent antiviral activity in a human infection model and expand the evidence to Q7D administration. Reducing the loading period from five to two days and transitioning from once-daily to once-weekly dosing are improved pharmacokinetic parameters which can improve ease of use if this drug is developed further.

## Supplementary Material

Supplementary appendix

## Figures and Tables

**Figure 1.**
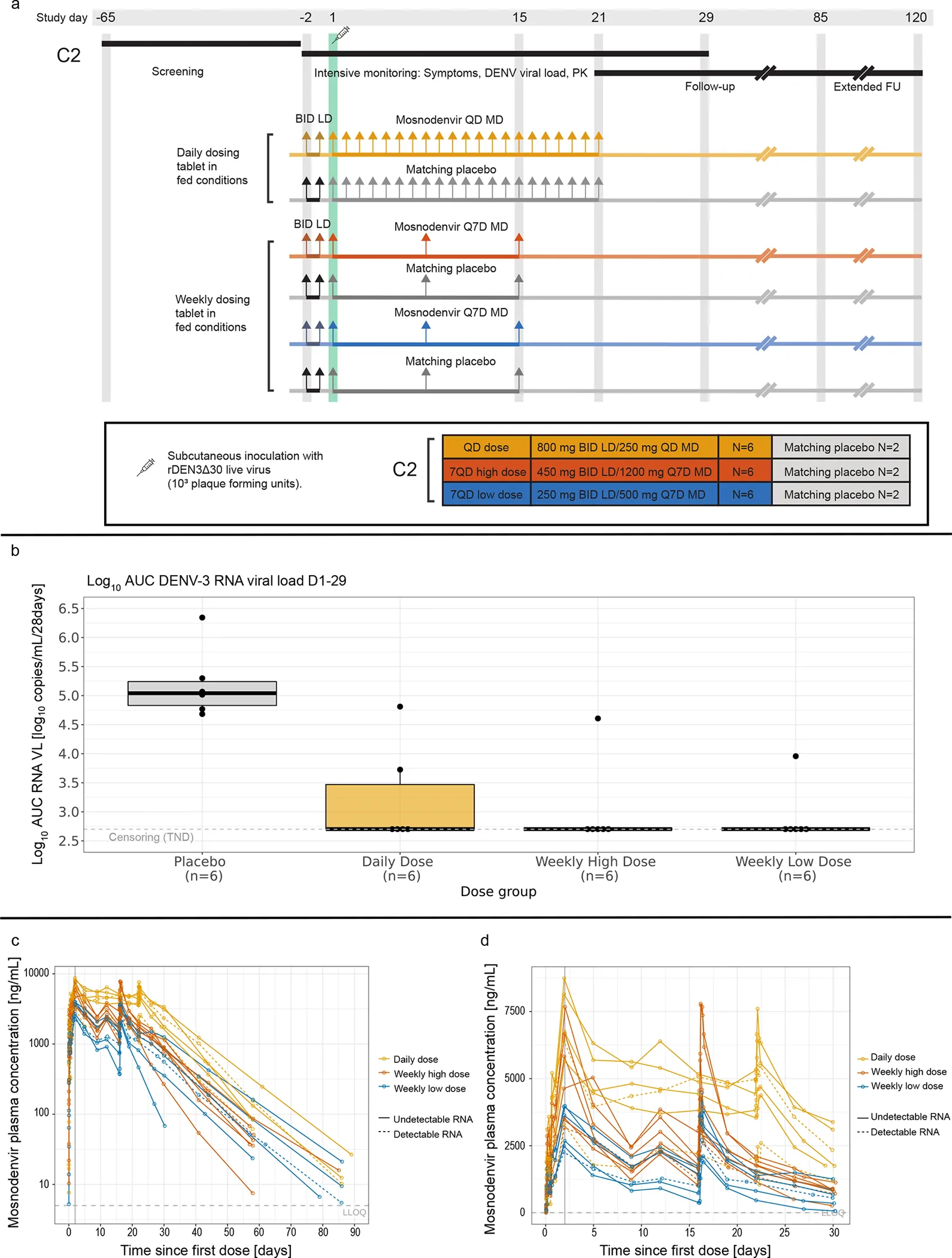
A. Study design for Cohort 2 BID, twice a day; C2, Cohort 2; DENV, dengue virus; FU, follow-up; LD, loading dose; MD, maintenance dose; PK, pharmacokinetics; QD, daily; Q7D, once a week, rDEN3Δ30, recombinant dengue serotype 3. B. DENV-3 RNA log_10_ AUC_D1-D29_ (VL) Solid black dots represent the individual log_10_ AUC_D1-D29_ (viral load) values. Box plots represent the 25^th^, 50^th^ and 75^th^ percentile. Dots at the censoring (target not detected, TND) line represent participants with undetectable DENV-3 RNA throughout D29. C and D. Individual mosnodenvir plasma concentration profiles versus time since first dose (PK analysis set) c, through Day 85 with concentrations on a log_10_ scale. d, through Day 29 with concentrations on a linear scale. Mosnodenvir plasma concentrations below the lower limit of quantification (LLOQ, 5.00 ng/mL) are not displayed. Solid lines indicate undetectable and dashed lines indicate detectable DENV-3 RNA at the participant-level. Solid vertical line indicates time of rDEN3Δ30 inoculation on Day 1. Dashed horizontal line indicates the LLOQ.
