# Supplementary material for: Once-Weekly Mosnodenvir as Dengue Prophylaxis in a Human Infection Model
Source: N Engl J Med. Author manuscript; Available in PMC 2026 Oct 15. (PMC13671277; doi:10.1056/NEJMc2516395)
Supplement: Supplementary file 1 [file NIHMS2203801-supplement-Supplementary_appendix.pdf]

Table of Contents

**Preamble** ... 2
**Methods** ... 3
***Study design*** ... 3
***Participants*** ... 3
***Study procedures*** ... 4
***Study objectives and sample size*** ... 4
***Virology, immunology and pharmacokinetic assessments*** ... 4
***Statistical analysis*** ... 5
**Additional results** ... 6
***Participants*** ... 6
***Daily dose regimen*** ... 6
***Weekly dose regimens*** ... 7
***Viral genome sequencing*** ... 7
***Safety*** ... 7
***Pharmacokinetics*** ... 8
**Concluding remarks** ... 9
**Supplementary figures** ... 11
**Supplementary tables** ... 16
**References** ... 25
**Acknowledgments and sources of fundings** ... 29
**Competing interest** ... 29

**Preamble**

Dengue has emerged as an expanding and accelerating threat to human health.[1] Record dengue fever surges have been reported internationally in 2024[1,2] and underscore the need for medical countermeasures. A small proportion of dengue cases progress to severe diseases such as dengue hemorrhagic fever or dengue shock syndrome, which are associated with a high fatality rate, particularly among children.[3] Two vaccines, CYD-TDV (Dengvaxia) and TAK-003 (Q-Denga) are currently approved for use.[4-6] Two other vaccines, Butantan-DV (Butantan Institute) and V181 (Merck) are currently under investigation[7-9] and Butantan-DV has been filed for licensure in Brasil.[10,11] Antiviral prophylaxis and treatment are currently not available for dengue and treatment consists mainly of supportive care.

Mosnodenvir (also known as JNJ-64281802) is a small molecule that belongs to a class of imidazopyridines and blocks dengue virus (DENV) replication by targeting the interaction between nonstructural viral proteins NS3 and NSB4.[12-14] Preclinical data demonstrate that mosnodenvir has potent antiviral activity against DENV.[13,14] In a first-in-human phase 1 study, mosnodenvir was administered as single doses (50-1200 mg) or multiple daily (QD) doses (50-560 mg for 10 days or 400 mg for 31 days).[15] Cohort 1 (C1) results of the current study have been recently published and demonstrate that QD doses of mosnodenvir reduced DENV-3 viral load versus placebo in a dose-dependent manner with a statistically significant difference observed between high-dose mosnodenvir and placebo and without relevant clinical safety findings in a CHIM.[16] Addition of a higher QD dose in Cohort 2 (C2) further supported the exposure-response assessment. The physicochemical properties of mosnodenvir (e.g., the terminal elimination half-life [$t_{1/2}$] of 6.3–9.2 days) provides the opportunity for less frequent dosing.[15] Weekly (Q7D) or twice weekly dosing of mosnodenvir in healthy participants was associated with an adequate pharmacokinetic profile and was without evident safety concerns.[17] Therefore, two Q7D dose regimens were investigated in C2 of the study reported in the **Letter to the editor**. Full details of the methods employed, additional results obtained, and a conclusion are included in this appendix.

## Methods

### *Study design*

This is a phase 2a, randomized, double-blind, placebo-controlled CHIM study conducted at the Johns Hopkins School of Public Health and the University of Vermont College of Medicine, both in the United States (US), starting in February 2022 (ClinicalTrials.gov NCT05048875). The study consisted of two cohorts conducted in a staggered manner. Different QD doses of mosnodenvir, provided in oral capsules and administered under fasted conditions, were investigated in C1.[16] In C2, one QD dose regimen, mosnodenvir 800 mg BID LD/250 mg QD MD, and two Q7D dose regimens, high-dose mosnodenvir 450 mg BID LD/1200 mg Q7D MD and low-dose mosnodenvir 250 mg BID LD/500 mg Q7D MD, were compared with placebo (**Figure 1A**). For C2, mosnodenvir was supplied as 50-mg, and 200-mg tablets administered under fed conditions. Matching placebo formulations were provided. The study was conducted in accordance with Good Clinical Practice guidelines and the Declaration of Helsinki. Written informed consent was obtained from each participant prior to any study-related activities. The study protocol (available with the full text of the letter at NEJM.org) and informed consent form were reviewed and approved by Independent Ethics Committees at each site. Data were gathered by the study site investigators and analyzed at Johnson & Johnson in collaboration with the National Institutes of Health (NIH). All the authors vouch for the accuracy and completeness of the data presented and for the fidelity of the study to the protocol. Medical writing assistance was funded by Johnson & Johnson.

### *Participants*

We enrolled healthy individuals 18–55 years of age, who were confirmed to be seronegative to DENV, Zika virus (ZIKV), West Nile virus, and Saint Louis encephalitis virus (SLEV) or did not have a history of vaccination for DENV, ZIKV, or Japanese Encephalitis virus (JEV) prior to enrollment, had not traveled to any dengue-endemic region within 4 weeks from enrollment nor planned to do so during the study, and had not received or would not receive any live attenuated vaccines within 28 days before or after study drug intake. For more details on the inclusion and exclusion criteria, see the **protocol**.

***Study procedures***

Participants attended screening between D−63 and D−3 and were randomized into three arms (one QD and two Q7D dosing arms) and further randomized to receive either mosnodenvir (N=6) or matching placebo (N=2). The LD was given from D-2 to D-1, followed by a QD MD from D1 to D21 or Q7D MD on D1, D8 and D15 ( high and low-dose arms), with a subcutaneous inoculation of 3 $\log_{10}$ plaque-forming units of the under-attenuated virus rDEN3Δ30[18] on D1 (**Figure 1A**). All participants were admitted to the inpatient unit for the first two dosing days (D-3 through D-1) and were observed for at least 30 minutes after initial dosing and/or inoculation to ensure their safety. Safety parameters and adverse events (AEs) were monitored throughout the study as previously described.[16] Blood samples were taken at regular time points for virology and pharmacokinetic assessments. Participants were initially followed up through D85. For C2, sampling timepoints were adapted to accommodate weekend days and the protocol was amended to add an additional follow-up visit at D120 to collect an extra blood sample for humoral immunity. As some of the participants had already completed the study at the time of the amendment, this visit window was extended to D360. This visit was considered a post-study visit for all participants. More information about the study procedures is available in the **protocol**.

***Study objectives and sample size***

The primary objective of this study was obtained by testing C1 QD high dose versus placebo and is described elsewhere.[16] C2 was intended to further characterize the relationship between pharmacokinetics and antiviral activity and to assess the feasibility of a weekly dose regimen. As this is an exploratory cohort, no formal sample size determination and power calculations were performed. Other secondary objectives included safety and tolerability, occurrence and severity of DENV infection-associated AEs, other virology parameters, pharmacokinetics of mosnodenvir.

***Virology, immunology and pharmacokinetic assessments***

Virology and pharmacokinetic assessments were obtained as described previously.[16] For immunology assessments, volunteers were screened for DENV, ZIKV, West Nile and SLEV neutralizing antibodies by the gold standard focus reduction neutralization test (FRNT) prior to enrollment. Participants were required to have an $FRNT_{50}$ <10 to each virus as evidence of lack of previous virus exposure. This test was performed within 60 days of enrollment. All participants had a blood sample drawn for antibody testing on Day -2 (prior to starting mosnodenvir and prior to DENV-3 challenge) at baseline. Additional details about these and other assessments are available in the **protocol**.

***Statistical analysis***

The safety analysis set is defined as all randomized participants (or their replacements) who received at least 1 dose of study drug. The efficacy analysis set is defined as participants from the safety analysis set who were inoculated with rDEN3Δ30 and without protocol deviations that could affect the antiviral activity assessment. Descriptive statistics are provided to support the assessment of the secondary objectives. Analyses were performed with SAS 9.04 (SAS Institute Inc., Cary, NC, USA). Graphs were created in R version 4.2.0 or higher (Comprehensive R Network, http://cran.r-project.org/).

## Additional results

### *Participants*

In Cohort 2, 25 participants were recruited between October 2023 and March 2024 and were included in the safety analysis set. 24 participants were randomized to receive either mosnodenvir (N=18) or placebo (N=6), 1 additional participant was enrolled as a replacement for a participant in the mosnodenvir Q7D high-dose group and 24 participants were included in the efficacy analysis set (**Figure 1A** and **Table S2**). All participants were 100% compliant to the study intake except for the replaced participant who missed the D15 MD intake but attended the remaining scheduled visits and who was excluded from the efficacy analysis set. Baseline characteristics were similar between the placebo and mosnodenvir arms (**Table S2**) and balanced across the different dosing arms, except for fewer females in the QD dose arm (17%) when compared to the other arms (**Table S2**). The representativeness of the study population is shown in **Table S3**.

### *Daily dose regimen*

The virology parameters (DENV RNA peak, duration and onset of first detectable DENV RNA) from the 6 new placebo participants in C2 were comparable with the placebo participants from C1 (**Figure S1**). The proportion of participants with undetectable DENV-3 RNA was 67% (4/6) in the QD dose arm, versus 0% (0/6) in the placebo arm (**Figure 1B and Figure S2**). In all mosnodenvir-dosed participants without detectable DENV-3 RNA, no anti-DENV IgM/IgG nor nAbs were observed until D120 (**Figure S2 and Table S4**). For the 2 participants with detectable DENV-3 RNA, the peak DENV-3 RNA levels were comparable or lower to placebo, but with a delayed onset (**Figure S2**), and positive anti-DENV IgM and/or IgG and/or nAbs values after baseline were observed (**Figure S2 and Table S4**). In one of those participants, infectious virus and a DENV-associated rash, defined as rash in combination with detectable DENV-3 RNA, were observed (**Figure S2**).

***Weekly dose regimens***

Eighty-three percent (5/6) participants in each of the Q7D regimens were without signs of DENV3 infection (undetectable DENV-3 RNA, no increase in anti-DENV IgM/IgG/nAbs) versus 0% (0/6) in the placebo arm (**Figure 1B, Figure S2 and Table S4**). Although 1 participant from the Q7D high dose missed the D15 dosing (and is therefore excluded from the efficacy analysis set), DENV-3 RNA and anti-DENV IgM/IgG/nAb levels remained undetectable up to D85. For the 2 participants with detectable DENV-3 RNA (one in each Q7D regimens), the peak DENV-3 RNA levels were comparable or lower to placebo, but with a delayed onset (**Figure S2**), and positive anti-DENV IgM and/or IgG and/or nAbs values after baseline were observed (**Figure S2 and Table S4**).

***Viral genome sequencing***

At least one emergent NS4B amino acid variation was detected in 100% (4/4) of participants with available NS4B sequencing data in the mosnodenvir dose arms, as compared to 33.3% (2/6) of participants in the placebo arm. (**Table S5**). The emergent NS4B variations included A19L (N=1), V91G (N=2), L94F (N=1), Y99H (N=1), with variant frequencies consistently >99%, in the mosnodenvir arms versus S60I (N=1) and I201V (N=1), with variant frequencies between 19-99%, in the placebo arm.

***Safety***

All participants reported at least one AE starting at first study drug intake until study termination (**Table S1**). Most AEs were mild (grade 1) to moderate (grade 2), and resolved without complications. All out-of-range laboratory findings were isolated and fully reversible. In the mosnodenvir arms, 2 (10.5%) participants reported a severe AE (grade 3), of which 1 was related to elevated liver function tests (D4) lasting 48 hours and 1 was a temporary aphasia and visual impairment (D62). A severe AE was reported in 3 participants in the placebo arm, all of which were related to laboratory abnormalities (grade 3). All reported severe AEs were unrelated to the study drug and/or the inoculation, as assessed by the principal investigator. None of the participants had serious AEs and none died (**Table S1**).

***Pharmacokinetics***

For both QD and Q7D dose regimens, mosnodenvir plasma concentrations rapidly increased with the BID LD from D-2 to D1 and were well maintained over the 21 days MD phase, after which concentrations declined slowly consistent with a long $t_{1/2}$ (6.8–11 days observed in C2 (**Table S6**), 7.5-11 days in C1[16] and 6.3–9.2 days in other studies[15,17]) (**Figures 1C and 1D**). One participant in the QD dose arm, who had approximately 2-fold lower exposures as compared to the other participants in the same arm, had detectable DENV-3 RNA with a placebo-like profile (**Figure 1D**, and **Figure S4**). Mosnodenvir exposures were generally higher for the Q7D high-dose as compared to the Q7D low-dose arm, however, individual concentration profiles did overlap between the two Q7D dose arms (**Figures 1C and 1D**). Pharmacokinetic parameters on D-2 and D21 (for QD dose arms) or D15 (for Q7D dose arms) are summarized in **Table S6**.

**Concluding remarks**

Mosnodenvir is an antiviral against DENV infection that has reached phase 2 clinical development in a prophylactic setting.[16] Initial results from this Phase 2a CHIM study were expanded with the investigation of additional QD and Q7D dose regimens in C2. With the inclusion of 6 additional placebo participants in C2, the robustness of this human infection model with 100% infection rate and similar virology profiles was confirmed, indicating that a limited number of participants could be required to adequately power efficacy studies to detect statistical significance. The findings established in C1 QD high-dose regimen (60% [6/10] without signs of DENV-3 infection [undetectable DENV-3 RNA; no anti-DENV IgM/IgG/nAbs [16]]) were confirmed, with 67% (4/6) of dosed participants in C2 QD dose arm without signs of DENV-3 infection **(Figure S1B**). Furthermore, QD administration of mosnodenvir in C1 low, medium and high dose and C2 QD dose arm resulted in a dose-dependent effect on the reduction of DENV3-RNA load (**Figure S1B**). Further evaluation of the concentration-effect relationship is ongoing including viral kinetic modeling.[19] The QD findings were expanded with 83% (5/6) participants without signs of DENV-3 infection in each of the C2 Q7D dose regimens. In C1 of the study, five days of QD loading doses (LD) under fasted conditions allowed mosnodenvir to reach steady state concentrations at the time of DENV-3 inoculation while in C2, BID LDs under fed conditions allowed the same in two days. Reducing the loading period to 2 days and shifting from QD to Q7D dosing may improve adherence from a convenience perspective. Intake of mosnodenvir under fed conditions increases plasma exposures compared to fasted conditions. Therefore, intake of mosnodenvir under fed conditions can support a less frequent (weekly) dose regimen.[20] The C2 weekly dosing results support further evaluation of weekly dosing in future development. Emerging genetic variations in NS4B were detected in all (4/4) participants with NS4B sequencing data available in the mosnodenvir arms, as compared to 33.3% (2/6) of the participants in the placebo arm. Similar to the C1 QD dose regimens,[16] the associated individual risk based on these limited data is considered minor, as no increase in the peak and duration of the DENV-3 RNA levels, nor in the frequency and severity of associated symptoms, was observed. Future field studies may offer valuable insights into the impact of those variations in contemporary virus strains.

There are limitations to the current study, such as the use of an under-attenuated DENV-3 challenge strain and the subcutaneous injection of healthy, seronegative participants. To generalize the current observations, the study findings must be confirmed in larger efficacy studies under field conditions, where multiple serotypes may co-circulate.[21] Additionally, transmission of the virus by the *Aedes* mosquito, as compared to subcutaneous injection may impact infection kinetics. Finally, in an endemic region, a high percentage of the population may have been previously exposed to DENV, with up to 90% seropositivity in hyperendemic regions.[22-25] Although different oral formulations of mosnodenvir in different food conditions were used in this study, a tablet in fed conditions in C2, versus a capsule formulation in fasted conditions in C1, mosnodenvir exposures were well maintained up to Day 21 with a low to moderate inter-subject variability. The tablet formulation was selected for further clinical development in a phase 2 field study.

To conclude, weekly dosing of mosnodenvir prevented signs of DENV-3 infection versus placebo without relevant clinical safety findings in this CHIM, supporting further investigation in future development.

## Supplementary figures

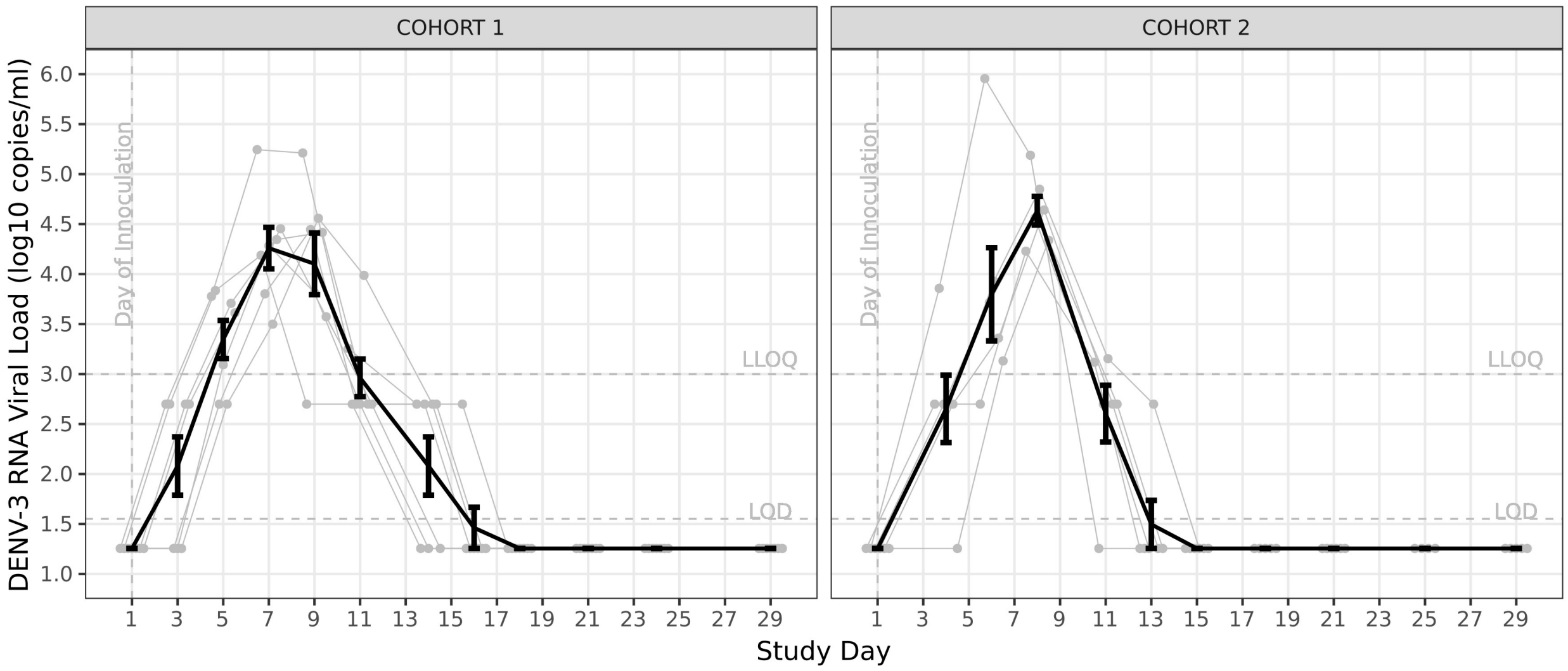


**Figure S1A. Individual virology profiles (DENV-3 RNA) and mean (+/-SE) over time - Placebo participants from Cohort 1 and 2**

Sampling timepoints for C1 included D1, 3, 5, 7, 9, 11, 14, 16, 18, 21, 23, 25, 29 .[16] Sampling timepoints for C2 included D1, 4, 6, 8, 11, 13, 15, 18, 21, 25, 29. Note: Sample days were updated in Cohort 2 to accommodate for operational challenges.

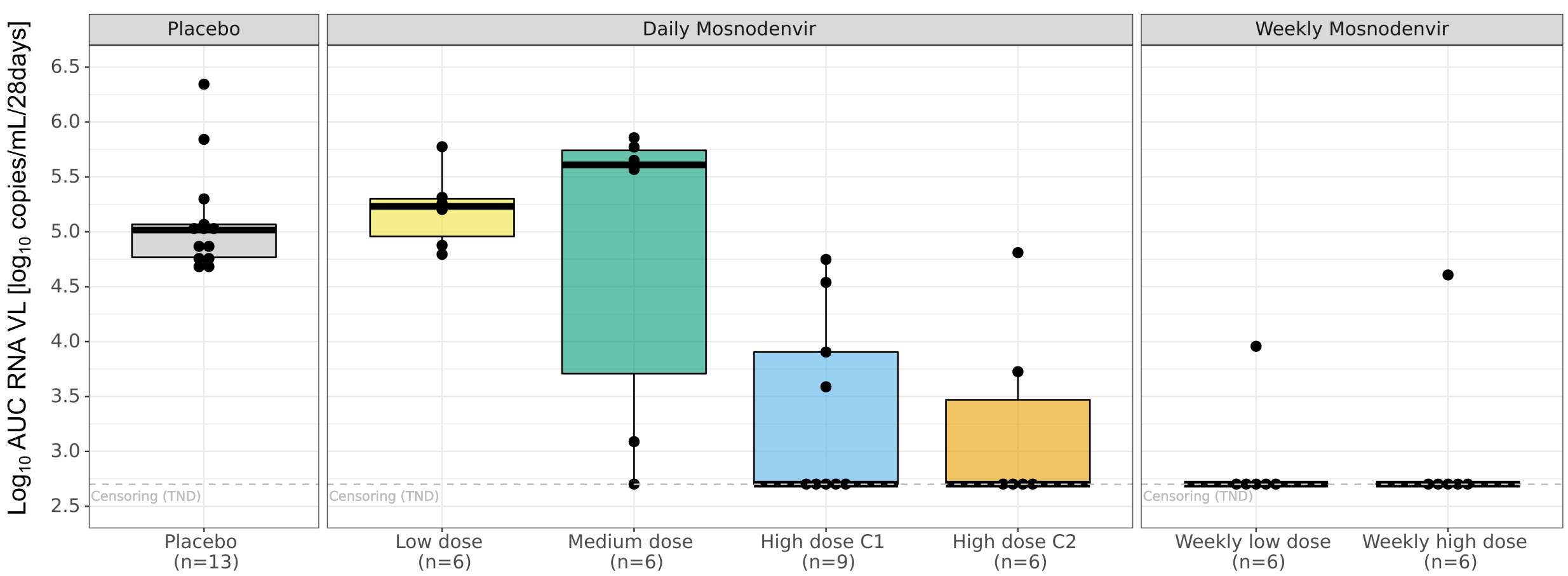


**Figure S1B. DENV-3 RNA $\log_{10}$ $AUC_{D1\text{-}D29}$ (VL) across all dosing regimens (Cohort 1 and 2)**

Solid black dots represent the individual $\log_{10}$ $AUC_{D1\text{-}D29}$ (viral load) values. Box plots represent the 25$^{th}$, 50$^{th}$ and 75$^{th}$ percentile. Dots at the censoring (target not detected, TND) line represent participants with undetectable DENV-3 RNA throughout D29. One participant in the C1 high-dose arm had a missing sample on D29 and was excluded from the primary analysis area under the DENV-3 RNA viral load concentration-time curves from D1 to D29 ($AUC_{D1\text{-}29}$ VL). Placebo participants from C1 and C2 are combined.

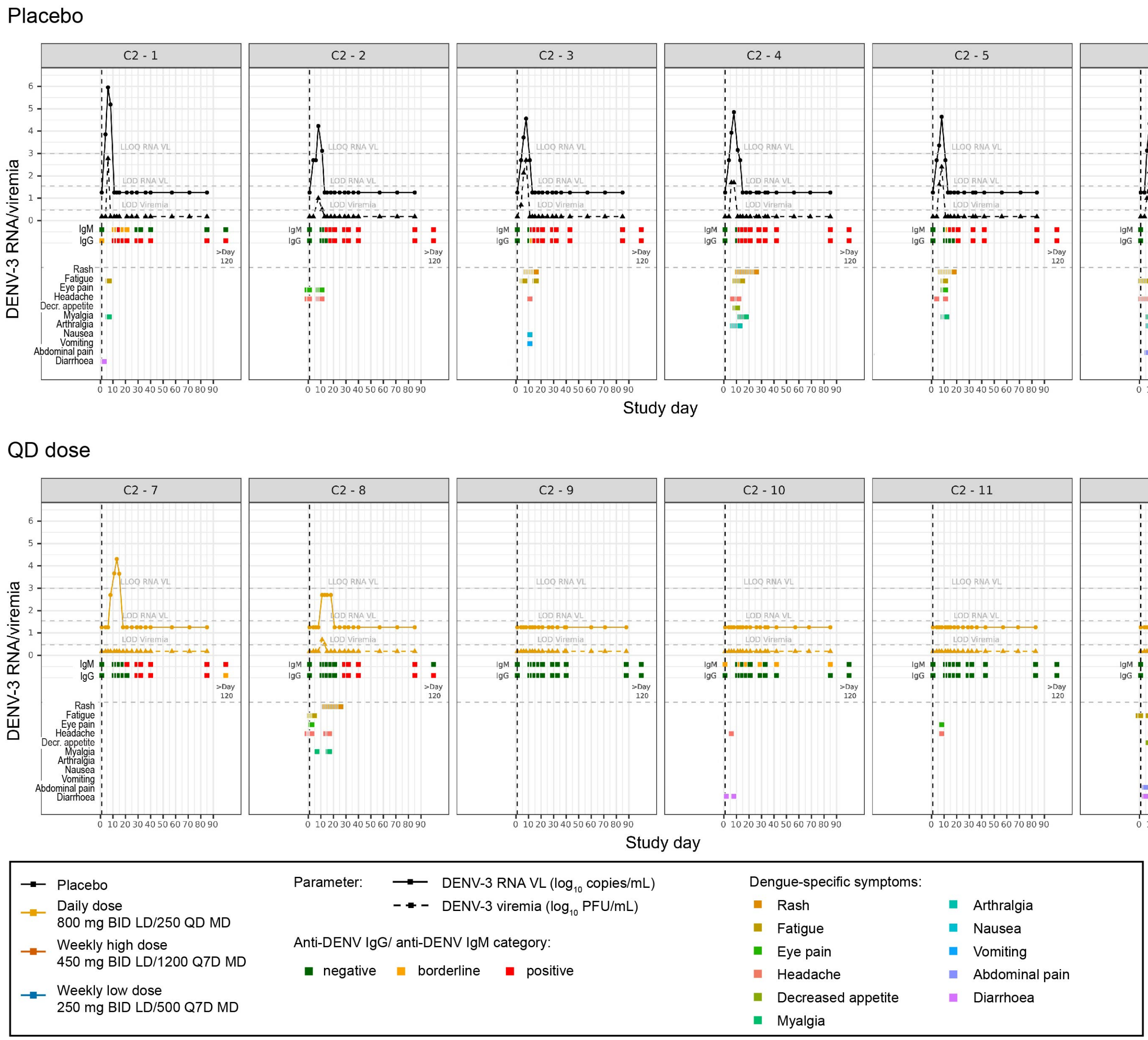
Placebo
C2 - 1
C2 - 2
C2 - 3
C2 - 4
C2 - 5
C2 - 6
QD dose
C2 - 7
C2 - 8
C2 - 9
C2 - 10
C2 - 11
C2 - 12
DENV-3 RNA/viremia
Study day
LLOQ RNA VL
LOD RNA VL
LOD Viremia
IgM
IgG
>Day 120
Rash
Fatigue
Eye pain
Headache
Decr. appetite
Myalgia
Arthralgia
Nausea
Vomiting
Abdominal pain
Diarrhoea
Placebo
Daily dose
800 mg BID LD/250 QD MD
Weekly high dose
450 mg BID LD/1200 Q7D MD
Weekly low dose
250 mg BID LD/500 Q7D MD
Parameter:
DENV-3 RNA VL (log10 copies/mL)
DENV-3 viremia (log10 PFU/mL)
Anti-DENV IgG/ anti-DENV IgM category:
negative
borderline
positive
Dengue-specific symptoms:
Rash
Fatigue
Eye pain
Headache
Decreased appetite
Myalgia
Arthralgia
Nausea
Vomiting
Abdominal pain
Diarrhoea

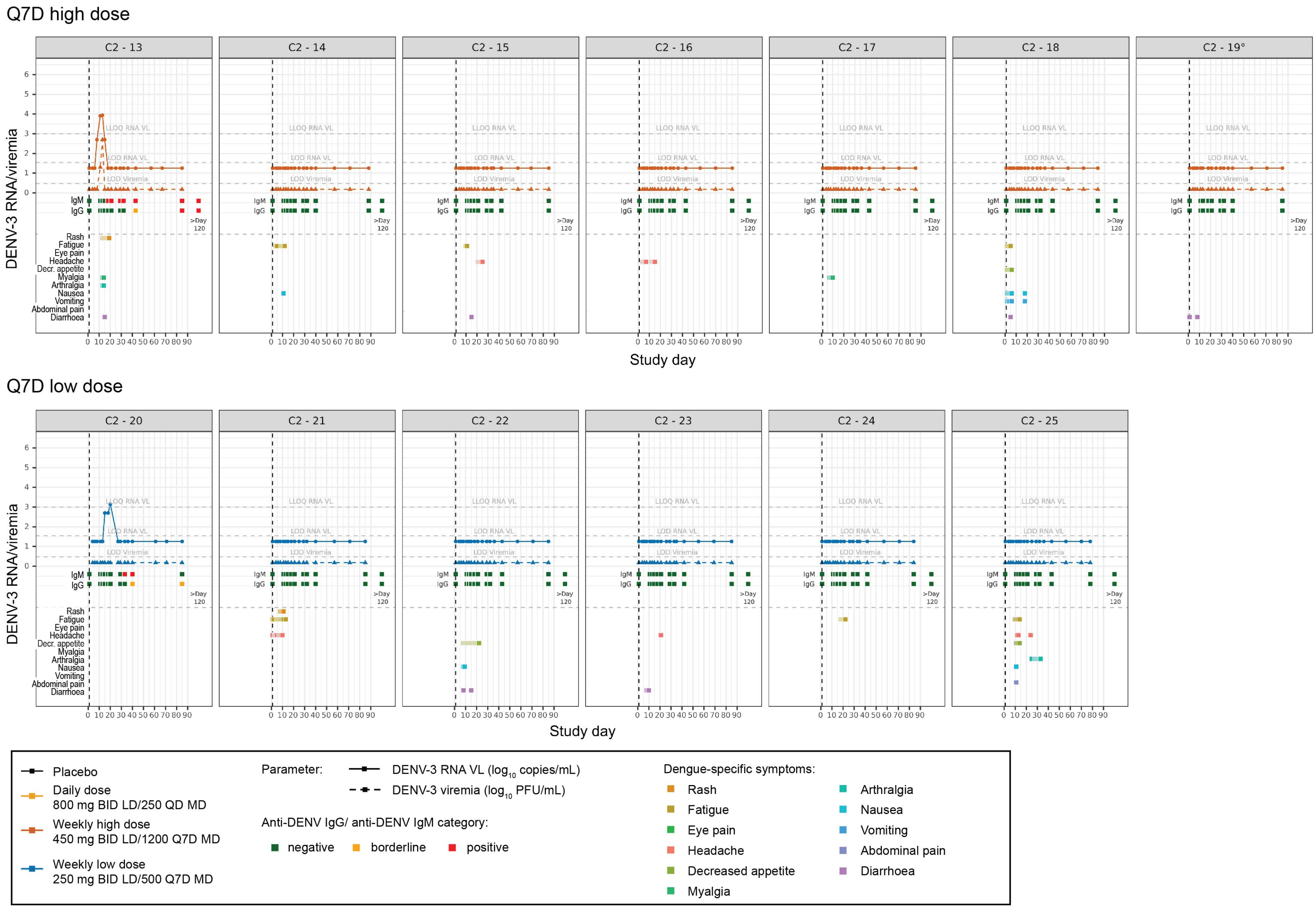


**Figure S2. Individual profiles of DENV-3 RNA, viremia, anti-DENV IgM/IgG and rash over time**

Participant C2-19 from the Q7D high dose missed the D15 dosing and was therefore excluded from the efficacy analysis set.

## Supplementary tables

# Table S1. Overview clinical safety data

**A: Adverse events from D-5 to D85 (Safety analysis set)**

| | Placebo N=6 | Mosnodenvir QD dose* N=6 | Mosnodenvir Q7D high dose* N=7 | Mosnodenvir Q7D low dose* N=6 | Mosnodenvir Combined N=19 |
|---|---|---|---|---|---|
| **Participants with at least 1 adverse event, n (%)**[†] | | | | | |
| Any | 6 (100.0) | 6 (100.0) | 7 (100.0) | 6 (100.0) | 19 (100.0) |
| Related to study treatment[‡] | 2 (33.3) | 1 (16.7) | 0 | 3 (50.0) | 4 (21.1) |
| Related to rDEN3Δ30 inoculation[‡] | 4 (66.7) | 0 | 3 (42.9) | 2 (33.3) | 5 (26.3) |
| Serious AEs | 0 | 0 | 0 | 0 | 0 |
| Leading to discontinuation of study treatment | 0 | 0 | 0 | 0 | 0 |
| Grade 1 as worst severity (mild) | 3 (50.0) | 5 (83.3) | 5 (71.4) | 4 (66.7) | 14 (73.7) |
| Grade 2 as worst severity (moderate) | 0 | 1 (16.7) | 0 | 2 (33.3) | 3 (15.8) |
| Grade 3 as worst severity (severe)[§] | 3 (50.0) | 0 | 2 (28.6) | 0 | 2 (10.5) |
| **Participants with the most common adverse events reported, n (%)**[‖] | | | | | |
| General Disorders And Administration Site Conditions | 1 (16.7) | 2 (33.3) | 3 (42.9) | 3 (50.0) | 8 (42.1) |
| Respiratory, Thoracic And Mediastinal Disorders | 0 | 2 (33.3) | 1 (14.3) | 4 (66.7) | 7 (36.8) |
| Metabolism And Nutrition Disorders | 2 (33.3) | 1 (16.7) | 4 (57.1) | 1 (16.7) | 6 (31.6) |

**B: Dengue infection-associated adverse events reported during challenge phase from D1 to D43 (Safety analysis set)**

| | Placebo N=6 | Mosnodenvir QD dose* N=6 | Mosnodenvir Q7D high dose* N=7 | Mosnodenvir Q7D low dose* N=6 | Mosnodenvir Combined N=19 |
|---|---|---|---|---|---|
| **Participants with at least 1 DENV infection-associated adverse event[¶], n (%)** | | | | | |
| Any | 6 (100.0) | 4 (66.7) | 7 (100.0) | 5 (83.3) | 16 (84.2) |
| Grade 1 as worst severity (mild) | 2 (33.3) | 2 (33.3) | 5 (71.4) | 5 (83.3) | 12 (63.2) |
| Grade 2 as worst severity (moderate) | 4 (66.7) | 2 (33.3) | 2 (28.6) | 0 | 4 (21.1) |
| **Participant with at least 1 DENV infection-associated adverse event[¶] by preferred term, n (%)** | | | | | |
| Headache | 5 (83.3) | 4 (66.7) | 2 (28.6) | 3 (50.0) | 9 (47.4) |
| Diarrhea | 1 (16.7) | 2 (33.3) | 4 (57.1) | 2 (33.3) | 8 (42.1) |
| Fatigue | 5 (83.3) | 2 (33.3) | 3 (42.9) | 3 (50.0) | 8 (42.1) |
| Nausea | 1 (16.7) | 1 (16.7) | 2 (28.6) | 2 (33.3) | 5 (26.3) |
| Decreased Appetite | 2 (33.3) | 1 (16.7) | 1 (14.3) | 2 (33.3) | 4 (21.1) |
| Eye Pain | 3 (50.0) | 3 (50.0) | 0 | 0 | 3 (15.8) |
| Myalgia | 4 (66.7) | 1 (16.7) | 2 (28.6) | 0 | 3 (15.8) |
| Rash Maculo-Papular | 4 (66.7) | 1 (16.7) | 1 (14.3) | 1 (16.7) | 3 (15.8) |
| Abdominal Pain | 1 (16.7) | 1 (16.7) | 0 | 1 (16.7) | 2 (10.5) |
| Arthralgia | 2 (33.3) | 0 | 1 (14.3) | 1 (16.7) | 2 (10.5) |
| Pyrexia | 1 (16.7) | 0 | 1 (14.3) | 0 | 1 (5.3) |
| Vomiting | 1 (16.7) | 0 | 1 (14.3) | 0 | 1 (5.3) |

[*] Actual treatment received (LD = loading dose; MD = maintenance dose; BID = twice daily; QD = daily; Q7D = weekly): QD dose: 800-mg BID LD/250-mg QD MD; Q7D high dose: 450-mg BID LD/1200-mg Q7D MD); Q7D low dose: 250-mg BID LD/500-mg Q7D MD.

[†] Solicited systemic AEs events reported from challenge up to and including the D43 visit are excluded from Table 2A as they are considered as DENV infection-associated AEs and are described in Table 2B. The headaches included in Table 2A are reported after Day 43.

[‡] Whether an adverse event was possibly related to mosnodenvir or to rDEN3Δ30 inoculation was determined by the principal investigator.

[§] Placebo: (1) decreased haemoglobin (D57–end of study), (2) increased lipase (D-1), (3) prolonged prothrombin time (D8–D11); C2 Q7D high dose: (1) increased alanine aminotransferase and AST (Day 4–6) during the challenge period, (2) aphasia and visual impairment (Day 62) in follow-up period.

[|] The most common adverse events were those that occurred in 4 or more participants who received mosnodenvir.

[¶] DENV infection-associated AEs are defined as solicited systemic AEs events reported from challenge up to and including the Day 43 visit. The challenge phase used is extended until visit Day 43, considering the half-life (~10 days) of the study intervention.

Participants were counted only once for any given event, regardless of the number of times they experienced the event. The event experienced by the participant with the worst severity was used. Adverse events are coded using the Medical Dictionary for Regulatory Activities Version 25.0. AE, adverse event. N, number of participants included in each arm; n, number of participants.

**Table S2. Analysis sets and baseline characteristics of study participants**

| | Placebo N=6 | Mosnodenvir QD dose* N=6 | Q7D high dose* N=7 | Q7D low dose* N=6 | Combined N=19 | Total N=25 |
|---|---|---|---|---|---|---|
| Randomized, n | 6 | 6 | 6 (+1 replacement)† | 6 | 18 (+1 replacement)† | 24 (+ 1 replacement)† |
| Received study drug, n | 6 | 6 | 7 | 6 | 19 | 25 |
| Inoculated with rDEN3Δ30, n (%) | 6 (100.0) | 6 (100.0) | 7 (100.0) | 6 (100.0) | 19 (100.0) | 25 (100.0) |
| Completed study drug, n (%)‡ | 6 (100.0) | 6 (100.0) | 6 (85.7) | 6 (100.0) | 18 (94.7) | 24 (96.0) |
| Completed study§, n (%) | 6 (100.0) | 6 (100.0) | 7 (100.0) | 6 (100.0) | 19 (100.0) | 25 (100.0) |
| Included in safety analysis set, n (%)ǁ | 6 (100.0) | 6 (100.0) | 7 (100.0) | 6 (100.0) | 19 (100.0) | 25 (100.0) |
| Included in efficacy analysis set, n (%)¶ | 6 (100.0) | 6 (100.0) | 6 (85.7) | 6 (100.0) | 18 (94.7) | 24 (96.0) |
| Included in PK analysis set, n (%)** | 0 | 6 (100.0) | 7 (100.0) | 6 (100.0) | 19 (100.0) | 19 (76.0) |
| Mean age (SD), years | 33.8 (12.11) | 29.2 (8.68) | 32.0 (8.58) | 29.0 (12.77) | 30.2 (9.64) | 31.0 (10.14) |
| Female sex, n (%) | 4 (66.7) | 1 (16.7) | 4 (57.1) | 4 (66.7) | 9 (47.4) | 13 (52.0) |
| Race, n (%)†† | | | | | | |
| N | 5 | 6 | 7 | 6 | 19 | 24 |
| Asian | 0 | 0 | 0 | 1 (16.7) | 1 (5.3) | 1 (4.2) |
| Black or African American | 2 (40.0) | 1 (16.7) | 2 (28.6) | 0 | 3 (15.8) | 5 (20.8) |
| White | 3 (60.0) | 5 (83.3) | 5 (71.4) | 5 (83.3) | 15 (78.9) | 18 (75.0) |
| Ethnicity, n (%)†† | | | | | | |
| N | 5 | 6 | 7 | 6 | 19 | 24 |
| Not Hispanic or Latino | 5 (100.0) | 6 (100.0) | 7 (100.0) | 6 (100.0) | 19 (100.0) | 24 (100.0) |
| Site, n (%) | | | | | | |
| N | 6 | 6 | 7 | 6 | 19 | 25 |
| John Hopkins University, Baltimore, MD | 3 (50.0) | 3 (50.0) | 4 (57.1) | 3 (50.0) | 10 (52.6) | 13 (52.0) |
| University of Vermont, Burlington, VT | 3 (50.0) | 3 (50.0) | 3 (42.9) | 3 (50.0) | 9 (47.4) | 12 (48.0) |

* Actual treatment received (LD = loading dose; MD = maintenance dose; BID = twice daily; QD = daily; Q7D = weekly): QD dose: 800-mg BID LD/250-mg QD MD; Q7D high dose: 450-mg BID LD/1200-mg Q7D MD); Q7D low dose: 250-mg BID LD/500-mg Q7D MD.

† Replacement assigned to the same study drug as participant who prematurely terminated study drug intake.

‡ Study Drug intake is considered completed if study drug was taken on Day 21 (QD dosing)/Day 15 (Q7D dosing), irrespective of missed doses before.

§ Study completion is intended as staying in the study up to Day 85; the follow-up extension up to week 120 is not included.

ǁ The safety analysis included all participants who received at least 1 dose of study drug.

¶The efficacy analysis included all participants from the safety population who were inoculated with rDEN3Δ30 and without major protocol deviations that could affect the antiviral activity assessment.

** The PK analysis included all participants with at least one available PK result after dosing (except placebo participants).

†† Race and ethnicity were reported by the participants.

N, number of participants included in each arm, reflecting non-missing values; n (%), number (percent) of participants, PK, pharmacokinetic; SD, standard deviation.

## Table S3. Representativeness of the study population

| Category | Assessment |
|---|---|
| Disease under investigation | DENV-associated infection |
| Special considerations related to | |
| Sex and gender | Some studies suggest that dengue is more common in males (≥15 years) than females. While more males than females present with milder disease, females account for more severe illness.[26,27] |
| Race or ethnic group | Some studies suggest that race and ethnicity may be a factor in the likelihood of infection and may also have an impact on dengue disease outcome. [28,29,30] |
| Geography | Dengue is endemic in tropical and sub-tropical climates worldwide, mostly in urban and semi-urban areas.[31,32] Limited local transmission has also been reported in southern Europe and the southern United States.[33,34] |
| Age | While the under 5 years of age group accounted for the largest portion of deaths and disability-adjusted life years in 1990, a shift toward those 15–49 years old was observed in 2019.[35] |
| Other considerations | Previous infection with DENV increases the risk of the individual developing severe dengue.[36] |
| Overall representativeness of this study | This study population consisted of healthy, DENV- and ZIKV- naïve non-pregnant adults 18–55 years of age, inclusive, recruited from the metropolitan Baltimore/Washington, DC area and Burlington, Vermont. The intent of a controlled human challenge model for DENV-3 infection is to induce measurable DENV-3 RNA levels and uncomplicated DENV infection-associated adverse events to support vaccine and drug development for the prevention and treatment of DENV infection. The results of this research cannot be extrapolated to the general population in dengue-endemic areas, but it provides valuable information on the potential antiviral activity of mosnodenvir in preventing dengue infection (in a dengue-naïve population that act as proxy for travelers to dengue-endemic regions as well as for naïve population living in dengue-endemic regions). |

DENV, dengue virus.

## Table S4. Detection of anti-DENV nAbs

| Dosing group | Subject ID | nAb titers | | | | |
|---|---|---|---|---|---|---|
| | | Study Day | | | | |
| | | -2 | 1 | 43 | 85 | 120 |
| C2 placebo | C2 - 1 | <10 | <10 | 16992 | 10927 | 1683 |
| | C2 - 2 | <10 | <10 | 6232 | 2847 | 2816 |
| | C2 - 3 | <10 | <10 | 6765 | 3205 | 3156 |
| | C2 - 4 | <10 | <10 | 2021 | 1462 | 718 |
| | C2 - 5 | <10 | <10 | 1526 | 714 | 550 |
| | C2 - 6 | <10 | <10 | 3272 | 1236 | 684 |
| C2 QD dose | C2 - 7 | <10 | <10 | 20480 | 5759 | 1445 |
| | C2 - 8 | <10 | <10 | 1839 | 615 | 219 |
| | C2 - 9 | <10 | <10 | <10 | <10 | <10 |
| | C2 - 10 | <10 | <10 | <10 | <10 | <10 |
| | C2 - 11 | <10 | <10 | <10 | <10 | <10 |
| | C2 - 12 | <10 | <10 | <10 | <10 | <10 |
| C2 Q7D low dose | C2 - 20 | <10 | <10 | 1653 | 863 | NA |
| | C2 - 21 | <10 | <10 | <10 | <10 | <10 |
| | C2 - 22 | <10 | <10 | <10 | <10 | <10 |
| | C2 - 23 | 21 | 16 | 16 | 14 | 19 |
| | C2 - 24 | <10 | <10 | <10 | <10 | <10 |
| | C2 - 25 | <10 | <10 | <10 | <10 | <10 |
| C2 Q7D high dose | C2 - 13 | <10 | <10 | 7747 | 1885 | 1040 |
| | C2 - 14 | <10 | <10 | <10 | <10 | <10 |
| | C2 - 15 | <10 | <10 | <10 | <10 | NA |
| | C2 - 16 | <10 | <10 | <10 | <10 | <10 |
| | C2 - 17 | <10 | <10 | <10 | <10 | <10 |
| | C2 - 18 | <10 | <10 | <10 | <10 | <10 |
| | C2 - 19 | <10 | <10 | <10 | <10 | NA |

yellow = participants with detectable DENV-3 RNA

red= detection of anti-DENV nAbs

°Participant C2-19 from the Q7D high dose missed the D15 dosing.

**Table S5. Comparison of prevalence of emerging amino acid genetic variations between placebo and mosnodenvir arm**

| | Placebo | Mosnodenvir | | | | Total |
|---|---|---|---|---|---|---|
| | | QD dose[1] | Q7D high dose[1] | Q7D low dose[1] | Combined | |
| Analysis set: inoculated, n | 6 | 6 | 7 | 6 | 19 | 25 |
| Number of participants with at least one sample with detectable DENV-3 RNA, n | 6 | 2 | 1 | 1 | 4 | 10 |
| Number of participants with at least one post-baseline sequencing result and inoculated virus sequence available | 6 | 2 | 1 | 1 | 4 | 10 |
| Any emerging genetic variations in NS4B | 2 (33.3%) | 2 (100.0%) | 1 (100.0%) | 1 (100.0%) | 4 (100.0%) | 6 (60.0%) |
| A19L | 0 | 0 | 1 (100.0%) | 0 | 1 (25.0%) | 1 (10.0%) |
| S60I* | 1 (16.7%) | 0 | 0 | 0 | 0 | 1 (10.0%) |
| V91G* | 0 | 1 (50.0%) | 1 (100.0%) | 0 | 2 (50.0%) | 2 (20.0%) |
| L94F* | 0 | 1 (50.0%) | 0 | 0 | 1 (25.0%) | 1 (10.0%) |
| Y99H* | 0 | 0 | 0 | 1 (100.0%) | 1 (25.0%) | 1 (10.0%) |
| I201V* | 1 (16.7%) | 0 | 0 | 0 | 0 | 1 (10.0%) |
| Any emerging genetic variations in NS4A° | 0 | 0 | 0 | 0 | 0 | 0 |
| Any emerging genetic variations in NS5 | 0 | 1 (50.0%) | 0 | 0 | 1 (25.0%) | 1 (10.0%) |
| G44R | 0 | 1 (50.0%) | 0 | 0 | 1 (25.0%) | 1 (10.0%) |
| V182A | 0 | 1 (50.0%) | 0 | 0 | 1 (25.0%) | 1 (10.0%) |
| Any emerging genetic variations in NS3 | 1 (16.7%) | 0 | 0 | 0 | 0 | 1 (10.0%) |
| P448L | 1 (16.7%) | 0 | 0 | 0 | 0 | 1 (10.0%) |
| Any emerging genetic variations in NS2A# | 0 | 0 | 0 | 0 | 0 | 0 |
| Any emerging genetic variations in NS2B° | 0 | 0 | 0 | 0 | 0 | 0 |
| Any emerging genetic variations in NS1# | 0 | 0 | 0 | 0 | 0 | 0 |
| Any emerging genetic variations in 2K | 1 (16.7%) | 0 | 0 | 0 | 0 | 1 (10.0%) |
| V18T | 1 (16.7%) | 0 | 0 | 0 | 0 | 1 (10.0%) |
| Any emerging genetic variations in E# | 0 | 0 | 0 | 0 | 0 | 0 |
| Any emerging genetic variations in M# | 0 | 0 | 0 | 0 | 0 | 0 |
| Any emerging genetic variations in ancC# | 0 | 0 | 0 | 0 | 0 | 0 |

| | Placebo | Mosnodenvir | | | | Total |
|---|---|---|---|---|---|---|
| | | QD dose[1] | Q7D high dose[1] | Q7D low dose[1] | Combined | |
| Any emerging genetic variations in pr[#] | 0 | 0 | 0 | 0 | 0 | 0 |

[1] Actual treatment received: QD dose: 800-mg BID LD/250-mg QD MD; Q7D high dose: 450-mg BID LD/1200-mg Q7D MD); Q7D low dose: 250-mg BID LD/500-mg Q7D MD.

[*] [‡] Only variations at NS4B positions of interest are shown. These were 30, 39, 43, 47, 60, 73, 85, 87, 88, 89, 91, 94, 99, 103, 104, 107, 108, 119, 135, 173, 179, 183, 190, 193, 195, 198, 201, 215, 216, 219, 222, 233, 237.

[#]Sequencing data for this fragment only available for 5 Placebo and 1 QD dose participant

°Sequencing data for this fragment only available for 6 Placebo, 1 QD and 1 Q7D low dose participant

Notes: Emerging genetic variation: absent, i.e., with a read frequency <3%, in the inoculated strain sequence but detected with a read frequency ≥15% at a post-baseline visit.

**Table S6. Pharmacokinetic parameters of mosnodenvir by treatment after first loading dose (Day -2) and last maintenance dose (Day 21 or Day 15)**

| (mean [SD], $t_{max}$: median [range]) | **QD dose: 800 mg mosnodenvir BID for 2 days, then 250 mg QD for 21 days** | **Q7D high dose: 450 mg mosnodenvir BID for 2 days, then 1200 mg Q7D for 15 days** | **Q7D low dose: 250 mg mosnodenvir BID for 2 days, then 500 mg Q7D for 15 days** |
|---|---|---|---|
| **Day -2** | | | |
| N | 6 | 7 | 6 |
| $C_{max}$, ng/mL | 2141 (1051) | 1894 (866) | 1187 (335) |
| $t_{max}$, h | 6.04 (4.02 - 8.23) | 8.00 (4.02 - 8.17) | 6.02 (4.00 - 8.03) |
| $AUC_{12h}$, ng.h/mL | 18499 (8962) | 16477 (6728) | 10047 (3258) |
| **Day 15/21**[a] | | | |
| N | 6 | 4[b] | 5[c] |
| $C_{trough}$, ng/mL | 4027 (1166) | 1500 (356) | 1228 (449) |
| $C_{max}$, ng/mL | 5245 (1745) | 5410 (1854) | 2826 (843) |
| $t_{max}$, h | 8.01 (4.00 - 12.07) | 4.01 (4.00 - 4.02) | 8.07 (4.00 - 12.03) |
| $AUC_{\tau}$, ng.h/mL | 113216 (35203) | 420271 (113827) | 263861 (88651) |
| $t_{½}$, h | 168 (28) | 164 (40) | 167 (57) |

[a] D15 refers to Q7D and D21 refers to QD dosing.

[b] n=6 for $C_{trough}$.

[c] n=4 for $C_{trough}$.

$AUC_{\tau}$, area under the concentration-time curve over the dosing interval; $AUC_{xh}$, area under the concentration-time curve from time 0 to x hours post-dose; BID, twice daily; $C_{max}$, maximum observed plasma concentration; $C_{trough}$, observed plasma concentration just prior to the beginning of a dosing interval; n, number of participants with evaluable data; QD, daily; Q7D, once a week; SD, standard deviation; $t_{max}$, time to reach the maximum observed plasma concentration; $t_{½}$, terminal elimination half-life.

**References**


1. World Health Organization. Dengue. Global burden. 21 August 2025 (https://www.who.int/news-room/fact-sheets/detail/dengue-and-severe-dengue).
2. World Economic Forum. The world is in the grip of a record dengue fever outbreak. What's causing it and how can it be stopped? 8 November 2024 (https://www.gavi.org/vaccineswork/world-grip-record-dengue-fever-outbreak-whats-causing-it-and-how-can-it-be-stopped).
3. Wong JM, Adams LE, Durbin AP, et al. Dengue: a growing problem with new interventions. Pediatrics 2022;149(6)
4. Malik S, Ahsan O, Mumtaz H, Tahir Khan M, Sah R, Waheed Y. Tracing down the updates on dengue virus-molecular biology, antivirals, and vaccine strategies. Vaccines (Basel) 2023;11(8):1328.
5. Centers for Disease Control and Prevention. Dengue. About a dengue vaccine. 15 May 2025 (https://www.cdc.gov/dengue/vaccine/index.html).
6. Cracknell Daniels B, Ferguson NM, Dorigatti I. Efficacy, public health impact and optimal use of the Takeda dengue vaccine. Nat Med 2025;31(8):2663-2672.
7. Kallas EG, Cintra MAT, Moreira JA, et al. Live, attenuated, tetravalent butantan-dengue vaccine in children and adults. N Engl J Med 2024;390(5):397-408.
8. Whitehead SS. Development of TV003/TV005, a single dose, highly immunogenic live attenuated dengue vaccine; what makes this vaccine different from the Sanofi-Pasteur CYD vaccine? Expert Rev Vaccines 2016;15(4):509-17.
9. Merck. News release. Merck Initiates Phase 3 Study Evaluating Dengue Vaccine Candidate. 12 June 2025 (https://www.merck.com/news/merck-initiates-phase-3-study-evaluating-dengue-vaccine-candidate/).
10. Folha de S Paulo. Butantan Institute Seeks Anvisa Approval for Single-Dose Dengue Vaccine. 17 December 2024

(https://www1.folha.uol.com.br/internacional/en/scienceandhealth/2024/12/butantan-institute-seeks-anvisa-approval-for-single-dose-dengue-vaccine.shtml).

11. Butantan Institute. Butantan pede à Anvisa registro da sua vacina contra a dengue, 1ª do mundo em dose única. 16 December 2024 (https://butantan.gov.br/noticias/butantan-pede-a-anvisa-registro-da-sua-vacina-contra-a-dengue-1-a-do-mundo-em-dose-unica?utm_source=twitter&utm_medium=social&utm_campaign=site-noticia&utm_term=site-dengue-anvisa&utm_content=site-dengue-anvis-registro).

12. Kesteleyn B, Bonfanti JF, Bardiot D, et al. Discovery of JNJ-1802, a first-in-class pan-serotype dengue virus NS4B inhibitor. J Med Chem 2024;67(5):4063-4082.

13. Kaptein SJF, Goethals O, Kiemel D, et al. A pan-serotype dengue virus inhibitor targeting the NS3-NS4B interaction. Nature 2021;598(7881):504-509.

14. Goethals O, Kaptein SJF, Kesteleyn B, et al. Blocking NS3-NS4B interaction inhibits dengue virus in non-human primates. Nature 2023;615(7953):678-686.

15. Ackaert O, Vanhoutte F, Verpoorten N, et al. Safety, tolerability, and pharmacokinetics of JNJ-1802, a pan-serotype dengue direct antiviral small molecule, in a phase 1, double-blind, randomized, dose-escalation study in healthy volunteers. Clin Infect Dis 2023;77(6):857-865.

16. Durbin AP, Van Wesenbeeck L, Pierce KK, et al. Daily mosnodenvir dosing as dengue prophylaxis in a human infection model. N Engl J Med 2025;393(21):2107-2118.

17. Kakuda TN, Harasym N, Buelens A, et al. Pharmacokinetics, safety, and tolerability of different maintenance dose regimens of mosnodenvir (JNJ-1802) in healthy adult participants. Clin Pharmacol Drug Dev 2025;14(12):960-970

18. Blaney JE, Jr., Hanson CT, Firestone CY, Hanley KA, Murphy BR, Whitehead SS. Genetically modified, live attenuated dengue virus type 3 vaccine candidates. Am J Trop Med Hyg 2004;71(6):811-21.

19. Goeyvaerts N, Woot de Trixhe X, Neyens M, et al. Viral kinetic modeling of mosnodenvir prophylaxis against DENV-3 in a controlled human infection model. Poster presentation. PAGE 33rd Meeting. Thessaloniki, Greece 2025.

20. Smith LE, D'Antoni D, Jain V, Pearce JM, Weinman J, Rubin GJ. A systematic review of factors affecting intended and actual adherence with antiviral medication as treatment or prophylaxis in seasonal and pandemic flu. Influenza Other Respir Viruses 2016;10(6):462-478.

21. Bos S, Zambrana JV, Duarte E, et al. Serotype-specific epidemiological patterns of inapparent versus symptomatic primary dengue virus infections: a 17-year cohort study in Nicaragua. Lancet Infect Dis 2025;25(3):346-356

22. Velandia-Romero ML, Coronel-Ruiz C, Castro-Bonilla L, et al. Prevalence of dengue antibodies in healthy children and adults in different Colombian endemic areas. Int J Infect Dis 2020;91:9-16.

23. Bonifay T, Le Turnier P, Epelboin Y, et al. Review on main arboviruses circulating on French Guiana, anultra-peripheric European region in South America. Viruses 2023;15(6):1268

24. Vicco A, McCormack C, Pedrique B, Ribeiro I, Malavige GN, Dorigatti I. A scoping literature review of global dengue age-stratified seroprevalence data: estimating dengue force of infection in endemic countries. EBioMedicine 2024;104:105134.

25. Nealon J, Bouckenooghe A, Cortes M, et al. Dengue endemicity, force of infection, and variation in transmission intensity in 13 endemic countries. J Infect Dis 2020;225(1):75-83.

26. Anker M, Arima Y. Male-female differences in the number of reported incident dengue fever cases in six Asian countries. Western Pac Surveill Response J 2011;2(2):17-23.

27. Guha-Sapir D, Schimmer B. Dengue fever: new paradigms for a changing epidemiology. Emerg Themes Epidemiol 2005;2(1):1.

28. de la CSB, Garcia G, Perez AB, et al. Ethnicity and difference in dengue virus-specific memory T cell responses in Cuban individuals. Viral Immunol 2006;19(4):662-8.

29. Halstead SB, Streit TG, Lafontant JG, et al. Haiti: absence of dengue hemorrhagic fever despite hyperendemic dengue virus transmission. Am J Trop Med Hyg 2001;65(3):180-3.

30. Blanton RE, Silva LK, Morato VG, et al. Genetic ancestry and income are associated with dengue hemorrhagic fever in a highly admixed population. Eur J Hum Genet 2008;16(6):762-5.

31. European Centre for Disease Prevention and Control. Dengue worldwide overview. July 2025 (https://www.ecdc.europa.eu/en/dengue-monthly).

32. Centers for Disease Control and Prevention. Areas with risk of dengue. 8 August 2025 (https://www.cdc.gov/dengue/areas-with-risk/index.html).

33. European Centre for Disease Prenvention and Control. Historical data on local transmission of dengue in the EU/EEA. 9 March 2025 (https://www.ecdc.europa.eu/en/all-topics-z/dengue/surveillance-and-disease-data/autochthonous-transmission-dengue-virus-eueea).

34. Center for Disease Control and Prevention. Public health considerations for dengue. 15 May 2025 (https://www.cdc.gov/dengue/php/public-health-considerations/index.html).

35. Yang X, Quam MBM, Zhang T, Sang S. Global burden for dengue and the evolving pattern in the past 30 years. J Travel Med 2021;28(8)

36. Halstead SB. Pathogenesis of dengue: dawn of a new era. F1000Res 2015;4

**Acknowledgments and sources of fundings**

This study was funded by the Intramural Research Program of the National Institute of Allergy and Infectious Diseases (NIAID) at the National Institutes of Health (NIH) and Johnson & Johnson. The study was performed under a Cooperative Research and Development Agreement with the National Institute of Health, an Agency of the U.S. Department of Health and Human Services. The contributions of the NIH author(s) were made as part of their official duties as NIH federal employees, are in compliance with agency policy requirements, and are considered Works of the United States Government. However, the findings and conclusions presented in this paper are those of the author(s) and do not necessarily reflect the views of the NIH or the U.S. Department of Health and Human Services. We thank the following Johnson & Johnson employees: Koen Ven for statistical programming, Caroline Ratky for data management, Erkki Lathouwers for virology monitoring, Yannick Vandendijck for statistical support and Tine De Marez and Annemie Hendrickx for their support in development strategy. The authors would like to thank Anne-Theres Henze for her medical writing support at Johnson & Johnson on behalf of Akkodis, Belgium. We thank all study participants, without whom this research would not have been possible and whose collaboration contributed to the advancement of health research in the field of tropical diseases.

**Competing interest**

Annemie Buelens, Kathleen Callewaert, Kim De Clerck, Veerle Van Eygen, Nele Goeyvaerts, Guillermo Herrera-Taracena, Thomas Kakuda, Marnix Van Loock, Sandra De Meyer, Freya Rasschaert and Liesbeth Van Wesenbeeck, are employees of Johnson & Johnson and may own stock or stock options in Johnson & Johnson. Oliver Lenz is a prior employee of Johnson & Johnson and may own stock or stock options in Johnson & Johnson and is a current employee of Glaxo SmithKline. Ruxandra Draghia Akli is a prior employee of Johnson & Johnson and may own stock or stock options in Johnson & Johnson and is a current employee of Novavax.
